# Preliminary Feasibility and Acceptability of a Culturally Specific Intervention for Reducing Sexual Revictimization of College Women

**DOI:** 10.15288/jsad.24-00024

**Published:** 2025-03-31

**Authors:** Kathleen A. Parks, Noelle M. St. Vil, Christopher Barrick, Sarah Ardalan, Robyn Lelito, Nicolette Kumkowski, Allyson Baio, Joame Lissade, Jenna Shaver, David Di Lillo

**Affiliations:** ^a^ Department of Psychology, State University of New York at Buffalo, Buffalo, New York; ^b^ School of Social Work, State University of New York at Buffalo, Buffalo, New York; ^c^ School of Nursing, State University of New York at Buffalo, Buffalo, New York; ^d^ Counseling, School and Educational Psychology, State University of New York at Buffalo, Buffalo, New York; ^e^ Department of Psychology, Queens College, New York, New York; ^f^ Department of Psychology, University of Nebraska at Lincoln, Lincoln, Nebraska

## Abstract

**Objective:**

More than 30% of women who experience sexual assault during college experience sexual revictimization (SRV) before graduating. Current sexual assault interventions have been developed with predominantly White samples, and most do not focus on reducing SRV or include effective alcohol reduction techniques. The purpose of the current study was to conduct a pilot randomized controlled trial to assess the feasibility and acceptability of a new intervention designed to reduce SRV in Black and White college women.

**Method:**

A sample of 59 women (*n* = 43, White; *n* = 16, Black) were randomly assigned to either the intervention or time and attention control condition. Both conditions consisted of two 90-minute in-person group sessions and two 30-minute online self-administered learning units. During the intervention, participants watched culturally specific videos (e.g., created in partnership with the cultural group, culturally congruent with regard to race of actors, vernacular, dress, and social situations) embedded with sexual assault risk cues. Women participated in discussions designed to improve risk recognition and assertive responses to sexual assault threats. All groups were racially homogeneous and had facilitators of the same race. Online intervention units included alcohol reduction strategies (e.g., personalized normative feedback) and safe dating practices.

**Results:**

Acceptability and feasibility of the intervention were good and suggested that cultural specificity was important for Black women. However, attrition was higher than expected, and barriers to participating were noted for Black women.

**Conclusions:**

These preliminary findings highlight the potential importance as well as the challenges in developing culturally specific sexual assault interventions for college women.

One in five women experience a sexual assault during college (Moylan et al., 2019; Muehlenhard et al., 2017), and many experience sexual revictimization (SRV) during this same time (30%–50%; Blayney et al., 2019; Norris et al., 2021). Both a history of sexual assault and heavy drinking, particularly heavy episodic drinking (HED; consuming ≥4 drinks on an occasion), are strong predictors of SRV (e.g., Humphrey & White, 2000; Mohler-Kuo et al., 2004; Parks et al., 2008c). However, many interventions do not address both predictors and the common ways they have been theorized to increase risk for SRV (i.e., decreased risk perception, less assertive responding). In addition, most college sexual assault interventions have been developed with predominantly White samples and lack sensitivity to cultural differences in risks for sexual assault among women of different races.

Using a stage model of behavioral therapy research (Rounsaville et al., 2001), we conducted a pilot randomized controlled trial (RCT) of the *Revictimization Prevention for College Women* (RPCW), designed to reduce college women's risk for SRV. This intervention is unique from other sexual assault interventions in two important ways. First, it focuses on decreasing risks for SRV by increasing recognition of sexual assault risk cues in social situations and decreasing heavy drinking. Second, it is culturally specific for both Black (e.g., African American, African, Afro-Caribbean, Afro-Latina, etc.) and White college women. Unlike non–culturally specific interventions, which rely on a one-size-fits-all approach, culturally specific interventions strive for congruence with regard to language, culture, and context (Gillum, 2008; Gutierrez et al., 2018; Heim & Kohrt, 2019) and are created in collaboration with the target population (Gillum, 2008). This is important because interventions designed and tested with one population (e.g., White women) and applied outside of that population (e.g., Black women) may not be effective (O'Brien & Macy, 2016). Thus, culturally specific interventions are crucial in ensuring intervention effectiveness (Gutierrez et al., 2018; O'Brien & Macy, 2016). We provide a brief overview of the theoretical underpinnings and research that informed the development of the content and cultural specificity of the intervention.

## Alcohol consumption

Alcohol consumption is involved in more than 50% of college sexual assaults (e.g., Abbey, 2002; Carey et al., 2015; Mohler-Kuo et al., 2004). With that said, individuals who experience sexual assault are never to blame for being assaulted, regardless of their surroundings, behavior, personal characteristics, or other factors. It is always the fault of the perpetrator for committing these acts of violence.

In a large study of college women, Walsh et al. (2014) found that women who had experienced sexual assault or SRV reported higher rates of monthly HED compared with women who had not experienced sexual assault. Parks et al. (2008b) used a daily diary study to determine that on days of HED, the odds of sexual assault (odds ratio [OR] = 19.4) were significantly higher than on nondrinking days. Experimental alcohol administration studies have found that women are less likely to notice sexual assault risk cues (e.g., isolation, sexual comments) and less likely to use assertive resistance (e.g., saying no, physically resisting) when intoxicated compared with sober controls (Davis et al., 2004, 2009; Parks et al., 2008a, 2016). These studies provide evidence for the association between alcohol consumption and sexual assault, as well as the impairing effects of alcohol intoxication that increase the risk for sexual assault. Therefore, decreasing incidents of intoxication should be a focus of sexual assault/SRV interventions.

## Prior sexual assault

Research indicates that a history of sexual assault is a strong predictor of SRV (Testa et al., 2010; Wilson et al., 1999). In a recent study of college women, Wiseblatt et al. (2025) found that having a precollege sexual assault was a significant predictor of sexual assault during the first semester at college for both White and Black women. Consequences of sexual assault that are thought to increase the risk for SRV include increased alcohol and drug use (McCauley et al., 2010; Palmer et al., 2010; Walsh et al., 2014) and decreased ability to perceive sexual assault risk cues (Messman-Moore & Brown, 2006; Wilson et al., 1999). Messman-Moore and Brown (2006) found that women who took longer to indicate that they would leave a hypothetical rape scenario reported higher rates of SRV at 8-month follow-up. In summary, both a history of sexual assault and alcohol intoxication decrease the likelihood that women will recognize a sexually threatening situation. Thus, interventions designed to reduce SRV must include alcohol reduction components (e.g., personalized normative feedback; Larimer et al., 2023; Miller et al., 2013) as well as risk recognition strategies.

## Cultural differences in sexual assault

The cultural context of sexual assault for Black women differs from that of White women. Sexual assault/SRV of Black women occurs within the context of racism and discrimination, misogynoir (misogyny toward Black women), and intersects with and heightens other significant social issues (e.g., poverty, housing insecurity), resulting in unique sexual assault experiences that adversely affect mental and behavioral health outcomes (Basile et al., 2016). Although the consequences of sexual assault among Black women may be like those of White women (e.g., substance use, risky sex), the cultural context in which their sexual assault exists and the increased likelihood of experiencing co-occurring public health issues are likely to magnify maladaptive behaviors and coping mechanisms in Black female sexual assault survivors (Basile et al., 2016).

Despite the differing sexual assault/SRV context of Black women, most intervention programs have been designed with samples that are a majority White. To date, findings on racial differences in rates of sexual assault/SRV and incapacitated rape are inconsistent. Some studies indicate that Black compared with White college women have higher rates and experience more severe sexual assaults (Coulter et al., 2017; Gross et al., 2006; Harris et al., 2021), as well as sexual assault by intimate partners (Lindquist et al., 2013; West & Johnson, 2013). However, several researchers have found that Black women are less likely to experience incapacitated sexual assault (Littleton et al., 2013; McQuiller Williams et al., 2016) and report less engagement in sex acts when they or their partner are intoxicated (McQuiller Williams et al., 2016) than White women. These findings have been explained by significantly lower drinking rates by Black compared with White college women (Barton et al., 2019). However, Krebs et al. (2011) found an association between the frequency of drinking and sexual assault among Black college women, indicating that alcohol use can be an important risk factor for sexual assault/SRV. Furthermore, a recent report on the health of underrepresented women found that HED rates among Black and White women over age 18 were similar (Office of Research on Women's Health, 2024).

The variation in rates and experiences of sexual assault/SRV among White and Black women points to the need for culturally specific interventions that consider the context of Black women's sexual assault experiences. However, we are unaware of any empirically supported sexual assault/SRV interventions targeting Black college women, despite findings from community samples that suggest culturally specific interventions are preferred (Gutierrez et al., 2018), increase accessibility (Heim & Kohrt, 2019), reduce service disparity (Hasnain et al., 2011), and maximize intervention effectiveness for Black populations (Gutierrez et al., 2018).

Recent research by Anderson et al. (2024) is focused on developing a culturally specific intervention to reduce sexual assault among Indigenous college students. To our knowledge, the RPCW would be the first culturally specific intervention developed to reduce SRV for Black college women.

## Current state of sexual assault interventions

Most sexual assault prevention programs have shown limited efficacy in reducing rates of sexual assault or SRV over an extended follow-up period or compared with an appropriate control (e.g., Ellsberg et al., 2015; Marx et al., 2001; Orchowski et al., 2008; Rothman & Silverman, 2007). We are aware of only one intervention that has shown efficacy over 12 to 24 months (Senn et al., 2015, 2017). Although Senn et al.'s (2015) intervention reduced primary sexual assault and SRV, it is not designed to address heavy drinking and is not culturally specific. We are aware of a small number of interventions that have focused on risk factors associated with SRV, such as posttraumatic stress disorder and alcohol consumption (e.g., Dworkin et al., 2024; Stappenbeck et al., 2021). To date, these interventions have focused on reducing these risk factors but not on demonstrated efficacy for reducing rates of SRV. We are not aware of any SRV interventions for college women that have been developed to increase risk perception of sexual assault cues, decrease heavy drinking, and are culturally specific for Black women.

## Current study

The purpose of the current study was to conduct a pilot RCT to assess the feasibility and acceptability of the *Risk Prevention for College Women* (RPCW), designed to reduce SRV in Black and White college women. The RPCW intervention was designed to be delivered over 1 week during two in-person sessions and two online units. We compared the RPCW to a time- and attention-matched health education control (HEC).

We developed and assessed a feasibility plan (i.e., recruitment, completion, retention) and acceptability (i.e., positivity toward intervention, safety of procedures) of the RPCW for both Black and White college women in preparation for conducting larger studies (Rounsaville et al., 2001). Given our small *N* and the preliminary nature of the RCT, we conducted exploratory analyses of the primary outcomes (i.e., heavy drinking and rates of SRV), which are included in Supplemental Materials.

## Method

### Participants and recruitment

The RCT was registered on clinicaltrials.gov (ID#: NCT05257603), and all procedures were approved by the university's institutional review board. We recruited women through emails sent to female undergraduates who provided information in the student directory; they were between ages 18 and 22 and self-reported as either White or Black. We also used fliers distributed widely, as well as culturally specific fliers (i.e., fliers with a picture of a Black woman on it and a bold heading [i.e., “Attention Black college Women!!!! We Need Your Help in Preventing Sexual Assault”]) targeting Black college women placed in strategic locations on campus (e.g., African Studies Program, Students of Color Student Union). Women were eligible to participate in the study if they were students at the university, had experienced a sexual assault since age 14, and reported HED at least once in the past 3 months. Women were excluded if they had a history of severe mental illness (i.e., bipolar disorder or schizophrenia) or current severe depression (score ≥ 31, Beck Depression Inventory–II; Whisman & Richardson, 2015), did not identify as heterosexual or bisexual, and/or were not sexually attracted to men.

**Figure 1.**
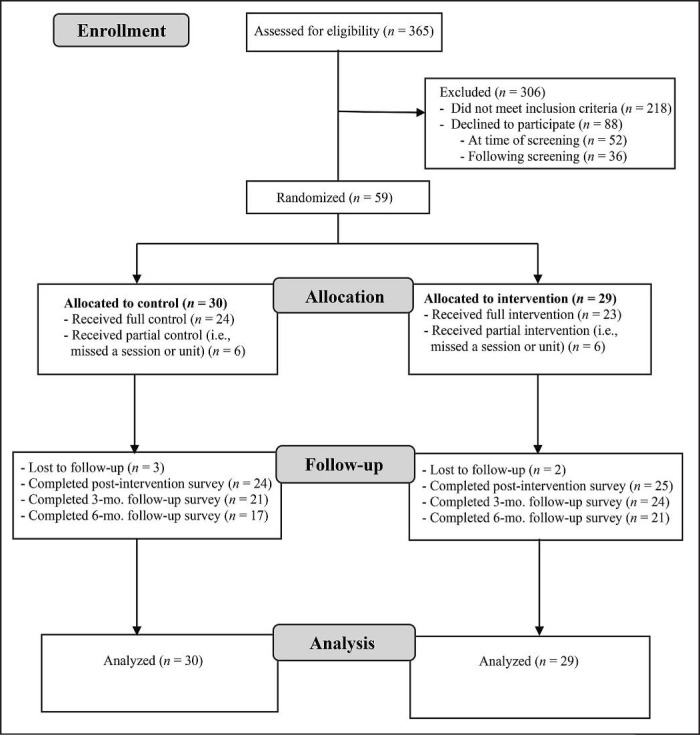
Flowchart of participants. Mo. = month.

Figure 1 provides a flowchart of participants. Of the women who contacted the study (*N* = 365), 24.1% (*n* = 88) declined to participate, 59.7% (*n* = 218) were ineligible, and 26% (*n* = 95) were eligible. The three primary reasons for ineligibility were race (*n* = 57, 30.5%), no HED in the past 90 days (*n* = 56; 29.9%), and no history of sexual assault (*n* = 33, 17.6%). Black women were more likely to be ineligible (57.4%, *n* = 27) for the study because of a lack of HED compared with White women (36.3%, *n* = 29), χ^2^(2) = 5.40, *p* = .02. Of those women who were eligible, 62% (*n* = 59) agreed to participate and were successfully randomized to condition (*n* = 29 [49.2%; *n* = 20 White, *n* = 9 Black] to RPCW; *n* = 30 [50.8%; *n* = 23 White, *n* = 7 Black] to HEC) and began the research protocol (i.e., completed baseline survey and attended first in-person session). The average age of participants was 19.5 years (*SD* = 1.3), 28.8% were freshmen, 20.3% were sophomores, and 25. 4% were juniors and seniors.

### Measures

*Screening instrument*.

- *Demographics:* Participants were asked to provide information on their individual characteristics (e.g., age, race).
- *Serious mental health conditions:* Women were asked to indicate whether they had ever been diagnosed with severe mental health conditions, including schizophrenia and bipolar disorder. The 54-item Beck Depression Inventory–II was used to assess current depression (Beck et al., 1996).
- *Sexual assault history:* A brief version of the Computer Assisted Maltreatment Inventory (DiLillo et al., 2010) was used to determine whether women had experienced adolescent sexual assault (i.e., age 14–18 years). Women were asked whether they had witnessed or experienced sexual activity, in the form of touching, being touched in a sexual way, and/or sexual intercourse: (a) “with anyone against your will or when you did not want it to happen”; (b) “with an immediate family member or other relative” (excluding similar age peers); or (c) “with anyone who was more than 5 years older than you” (excluding voluntary activities with a dating partner), when the participant was younger than age 18. The modified Sexual Experiences Survey (Koss et al., 1987; Testa et al., 2004) was used to determine whether women had experienced sexual assault (i.e., unwanted contact through completed rape by force or incapacitation) since age 18. Participants were asked to provide the date of the incident on which each endorsed Sexual Experiences Survey item had occurred. This allowed us to determine whether the participant experienced multiple forms of sexual assault within a single incident (i.e., sexual coercion as well as completed rape). The Computer Assisted Maltreatment Inventory and Sexual Experiences Survey are both behaviorally based measures with good psychometric properties for assessing sexual assault histories (DiLillo et al., 2010; Koss & Gidycz, 1985).
- *Heavy drinking:* The number of days of HED over the past 3 months was assessed using a modified item from the Daily Drinking Questionnaire (Collins et al., 1985). The item asked participants to indicate “on how many days in the past 3 months they consumed 4 or more drinks on a single occasion.” Women had to report 1 or more days of HED to be eligible for the study.

*Intervention acceptability: The Client Satisfaction Questionnaire*. The Client Satisfaction Questionnaire (Attkisson & Greenfield, 1999) is an eight-item measure that was used to assess overall satisfaction with the intervention (e.g., “In an overall, general sense, how satisfied are you with the program you received”). Each of the items is assessed on a 4-point scale. Average scores greater than 3 were considered the benchmark for good acceptability. Additional items were developed specifically for the current project to assess the acceptability of the in-person group sessions (four items) and the online units (four items). Individual items asked women to indicate how much they “enjoyed,” “learned new information,” “found the in-person sessions/online units interesting,” “learned valuable skills/gained information that I will use in the future,” and “found the in-person/online units unappealing (reverse scored).” These items were each rated on a 10-point scale (1 = *not at all* to 10 = *extremely*). An average score ≥ 7 on each item and for the overall scale was considered the benchmark for good acceptability. These measures were included in the post-intervention survey. Participants were e-mailed a link to access the post-intervention survey the day after they had completed their second in-person session (i.e., after intervention completion).

### Feasibility plan

In line with the stage model of intervention development, we developed a plan for assessing the feasibility of the intervention (Rounsaville et al., 2001). This included piloting our procedures for recruitment, screening, randomization, and assignment to condition, as well as procedures for training facilitators, monitoring fidelity, and adherence to intervention protocol. In addition, before the pilot RCT, we specified inclusion/exclusion criteria and the a priori analysis plan. A plan for monitoring safety (i.e., complaints, adverse/serious adverse events) and an email account for participants to communicate any concerns to the project were established. Feasibility was also assessed based on the success of recruitment and retention in the intervention protocol.

### Procedure

This was a pilot randomized intervention trial with a time- and attention-matched control. Both the intervention and control conditions involved women attending two (90-minute) in-person group sessions, 1 week apart, at the university (with a maximum of five other women) and completing two (30-minute) online units individually during the subsequent week. Eligibility screening, obtaining informed consent, and random assignment to conditions all occurred by telephone. Following assignment to a condition, women were scheduled for their in-person group sessions, emailed a link to the baseline survey, and asked to complete the survey before attending their first session. They received a reloadable bank card with $30 remuneration for the baseline survey at the first in-person session and three additional remunerations on the same bank card for the post-intervention ($30), 3-month ($40), and 6-month follow-up surveys ($50). Links to all follow-up surveys were sent via email. Confirmation emails and reminder texts were sent to remind women to complete each survey. Before leaving the second group session, women were assessed for concerns or questions and provided with a list of local and university resources.

### Study design

(A)*Intervention:* The RPCW in-person group sessions focused on increasing recognition of situations and behaviors that signal risk for SRV through presentation and discussion of videos that were culturally specific to Black and White college women. Two different sets of four videos, one for Black and one for White college students, were created in collaboration with each target population following an iterative process using focus group discussions (Parks et al., 2021; St. Vil & Parks, 2022). Examples of two videos are provided in the Supplemental Materials. The videos included different scenarios for each target population that incorporated cultural norms, expectations, and attitudes through specific language, situations, and settings described, developed, and refined during the focus group discussions. For example, Black women described attending house parties where the furniture was pushed against the walls so attendees could dance. They described parties consisting of mostly Black people, where male partygoers were “dressed to impress.” White women described attending “frat” parties that usually occurred in dark basements with sticky floors. These parties consisted of a majority White population, and women reported wearing crop tops, jeans, and old sneakers that could get ruined, whereas male attendees dressed in jeans or sweatpants and t-shirts.

The in-person sessions were racially homogeneous and led by a same-race facilitator. The first session began by showing women culturally specific videos depicting typical social situations (e.g., house/fraternity parties, concerts) embedded with sexual assault risk cues (e.g., unsolicited touching, isolation from friends, pressure to drink). Women then participated in a discussion about the sexual assault risk cues in each video and the emotions they experienced while watching the videos. The second in-person session integrated the online units with the initial in-person session, reviewed the videos and risk cues, and continued with a discussion of barriers to and techniques for responding in similar social situations. The session ended with a behavioral rehearsal of assertive responses to sexual threats and drinking pressure.

The online units focused on safe drinking and safe dating practices. Unit 1 provided women with personalized normative feedback about their drinking, National Institute on Alcohol Abuse and Alcoholism safe drinking guidelines, and drinking protective behavioral strategies. Unit 2 focused on dating protective behavioral strategies and respect in relationships. Personalized normative feedback and protective behavioral strategies have been used successfully in previous online interventions to reduce heavy drinking (Larimer et al., 2023; Miller et al., 2013) and have shown some preliminary efficacy in reducing sexual assault and risky sexual behavior (Gilmore et al., 2015; Lewis et al., 2014).

(B)*Time and attention control:* The HEC was a time- and attention-matched control to the RPCW. The two in-person sessions focused on reducing stress and improving sleep, whereas the online units focused on healthy eating and increasing physical activity. At the end of the 6-month followup, women in the HEC condition were emailed information about the purpose of the study and offered the option of receiving the RPCW intervention. No one from the HEC condition requested the RPCW.

*Intervention training and fidelity monitoring*. Group facilitators were master's-level students and above. All research staff were trained in all study protocols (e.g., screening, consent, randomization). Facilitator training included coaching in leading discussions about the videos (i.e., risk cue perception, response barriers, cognitive restructuring, and behavioral practice techniques). All group sessions were audio-recorded, and a research assistant completed a fidelity checklist during the session. The session recordings were reviewed later for fidelity of the protocol using the checklist by a second research assistant who was not present during the group session. The two checklists were compared to confirm fidelity, and the principal investigator assessed the consistency of 10% of the audio recordings and checklists.

*Data analysis*. Descriptive analyses of acceptability and feasibility measures were conducted with the intent-to-treat sample (*n* = 59).

## Results

### Feasibility

Recruitment, screening, and intervention procedures were delivered safely (i.e., no complaints or adverse events) per the planned pilot RCT protocol. Among the women who were randomly assigned to a condition, 79.3% (*n* = 23) completed the full RPCW intervention condition, and 80% (*n* = 24) completed the full HEC condition (i.e., both group sessions and both online units; Figure 1). Attrition (i.e., individuals not completing follow-up assessments) increased from post-intervention (HEC: 20%; RPCW: 13.8%) to 3-month (HEC: 30%; RPCW: 17.2%) and 6-month followup assessments (HEC: 43%; RPCW: 27.6%). The increase in attrition over the course of the study was not significantly different based on condition, χ^2^(2, *n* = 59) = 0.05, *p* = .98.

### Acceptability

Total scores for the Client Satisfaction Questionnaire, as well as overall satisfaction with the online and in-person components of the intervention by race and condition, are presented in Table 1. There was a nearly significant interaction between intervention condition and race for in-person session acceptability such that Black women rated the RPCW sessions higher than the HEC sessions, whereas White women rated the HEC sessions higher than the RPCW sessions, *F*(1, 45) = 5.78, *p* = .07. The average scores indicate that participants found the overall intervention and both the in-person and online components acceptable.

**Table 1.**
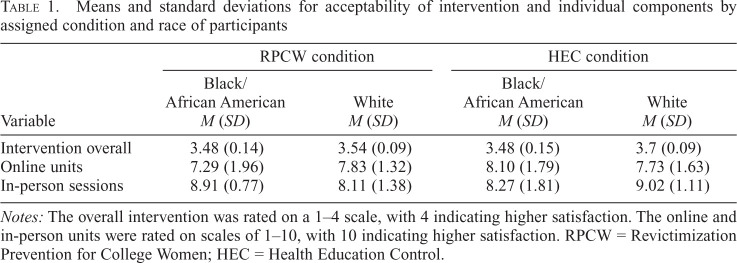
Means and standard deviations for acceptability of intervention and individual components by assigned condition and race of participants

Variable	RPCW condition		HEC condition	
	Black/African American *M* (*SD*)	White *M* (*SD*)	Black/African American *M* (*SD*)	White *M* (*SD*)
Intervention overall	3.48 (0.14)	3.54 (0.09)	3.48 (0.15)	3.7 (0.09)
Online units	7.29 (1.96)	7.83 (1.32)	8.10 (1.79)	7.73 (1.63)
In-person sessions	8.91 (0.77)	8.11 (1.38)	8.27 (1.81)	9.02 (1.11)

*Notes:* The overall intervention was rated on a 1–4 scale, with 4 indicating higher satisfaction. The online and in-person units were rated on scales of 1–10, with 10 indicating higher satisfaction. RPCW = Revictimization Prevention for College Women; HEC = Health Education Control.

## Discussion

The purpose of this pilot RCT was to assess the preliminary feasibility and acceptability of the RPCW. We developed the RPCW as a unique culturally specific intervention to reduce Black and White college women's risk for SRV. To our knowledge, no other college interventions have targeted preventing SRV by including culturally specific components and efficacious techniques for reducing heavy drinking and improving sexual assault risk perception.

Assessment of acceptability and feasibility indicated that the RPCW was rated above the threshold for both. We found no differences in acceptability based on race; however, Black women rated the in-person RPCW sessions somewhat higher than the in-person HEC units. This finding is noteworthy given that the RPCW in-person sessions, unlike the HEC, included the culturally specific videos developed for Black women and were implemented by Black group facilitators. This finding is like that of Gillum (2008), in which Black female participants in a culturally specific intimate partner violence intervention reported numerous benefits of the intervention and expressed appreciation of the cultural specificity. Our research provides some evidence that the culturally specific videos and in-person sessions with same-race facilitators resonated with participants. This pilot RCT was not fully powered to detect significant interaction effects. However, a fully powered efficacy trial might provide the opportunity to detect differences in acceptability based on race and more definitive evidence to support the need for culturally specific sexual assault/SRV interventions.

Assessment of feasibility indicated that procedures for recruitment, screening, and implementation of the protocol were successful. However, overall attrition was higher than anticipated, given the methods used to retain participants (i.e., reminder texts, phone calls, and increasing remunerations). This suggests that additional methods may be needed to keep women engaged for extended periods (e.g., >3 months). We also were unable to recruit the total number of Black women initially projected for the study (i.e., 1/3 of the sample). There are at least three possible reasons for this—first, the university has a low percentage of Black undergraduate students (<9%). Second, phone calls to Black participants who did not attend the full RPCW protocol revealed that they had other obligations that prevented their participation. This is supported by statistics suggesting that Black college students have more outside-of-school responsibilities (e.g., caregiving, full-time work) compared with students from other racial groups (Lloyd & Brown, 2023). Third, our inclusion criteria required that women report 1 day of HED in the past 3 months. There is research suggesting that Black college women drink less than White college women (Barton et al., 2019), and some evidence suggests that Black college women experience less alcoholfacilitated sexual assault (i.e., Littleton et al., 2013).

Recruitment of Black college women could be improved in several ways in future studies. First, more diverse universities should be included in larger multisite studies of the efficacy of this intervention. Given that Black college women appear to have more out-of-school responsibilities, greater flexibility and benefits should be considered to make participation in group sessions more appealing or easier. For example, offering women the possibility of remote participation through Zoom might be an option. This limits participants' need for transportation and often for childcare. In several previous studies, Zoom has successfully boosted participation in focus group discussions (Parks et al., 2021; St. Vil & Parks, 2022). Providing greater flexibility in group session times (i.e., mornings, evenings, and weekends) and transportation to sessions (e.g., Uber rides) could increase ease of attending in-person sessions. Setting up childcare options during group sessions or providing extra incentives to compensate for childcare costs might alleviate some of these additional responsibilities.

Finally, although Black women were more likely to be ineligible for the current study because of lower rates of HED than White women, we do not believe that this suggests that the RPCW will not be effective in reducing SRV among Black women who do not drink heavily frequently. Black women who participated in our study had to have at least one HED episode within the past 3 months to be eligible for the study. Therefore, it will be important in future studies to assess women with different patterns of heavy drinking to determine the efficacy of the RPCW. During the focus groups with Black college women used for the development of the in-person sessions, participants described socializing in settings that included heavy drinking, regardless of whether they were the ones consuming the alcohol (Parks et al., 2021; St. Vil & Parks, 2022). Research indicates that perpetrator alcohol use and drinking contexts (i.e., parties, bars) increase the risk for sexual assault (e.g., Abbey et al., 2022; Cleveland et al., 2019). Thus, it is likely that Black women are at higher risk for sexual assault when socializing in contexts with intoxicated others. Participation in the RPCW, which includes information on drinking and dating protective behavioral strategies that can be used in social drinking settings, might increase women's awareness of the risks of heavy drinking around them and thus decrease the risk for SRV. This needs to be tested in studies with larger samples to determine whether this differs based on race.

There are limitations to the current study. As indicated, this was a pilot RCT designed to assess the feasibility and acceptability of a new intervention. Although we did find that the intervention protocol was feasible and the intervention content was acceptable, we had difficulty recruiting the number of Black women we intended. Our attrition rate was higher than anticipated over the 6-month follow-up as well. Thus, our findings should be viewed with caution and used as a preliminary point for learning how to develop culturally specific sexual assault/SRV interventions that incorporate alcohol intervention techniques. We were not fully powered to detect statistically significant differences in our primary outcome measures; therefore, we need to conduct a larger, more rigorous Stage II efficacy trial to assess both primary and secondary outcomes.

Finally, there are differences in heavy drinking between Black and White women; therefore, although alcohol has been strongly associated with SRV, decreasing heavy drinking may not be an equally efficacious mechanism for reducing the risk for SRV in both Black and White women. Therefore, exploring the mechanisms that work for women of different races to reduce sexual assault/SRV should be an ongoing goal as we continue to develop culturally specific interventions.

## Conflict-of-Interest Statement

The authors have no conflicts of interest to disclose.
